# Remdesivir administration for Japanese COVID-19 patients undergoing maintenance hemodialysis: a retrospective observation with six case reports

**DOI:** 10.1186/s41100-022-00404-9

**Published:** 2022-04-11

**Authors:** Jun Ito, Moritsugu Kimura, Tomoyuki Toya, Konomi Isozumi, Atsuro Kawaji, Yudai Isozaki, Masafumi Fukagawa

**Affiliations:** 1 Kidney Center, Division of Nephrology and Diabetes, Tokai University Oiso Hospital, 21-1, Gakkyo, Oiso, Naka-gun, Kanagawa 259-0198 Japan; 2 Division of Nephrology and Metabolism, Department of Internal Medicine, Tokai University School of Medicine, 143, Shimokasuya, Isehara, Kanagawa 259-1193 Japan

**Keywords:** Novel coronavirus disease (COVID-19), Hemodialysis, Remdesivir, Sulfobutylether-beta-cyclodextrin (SBECD), Safety, Japanese

## Abstract

**Background:**

The mortality rate of novel coronaviral disease (COVID-19) patients undergoing dialysis is considerably higher than that of patients with normal kidney function. As of August 2021, only remdesivir has been approved in Japan as an antiviral drug for the treatment of COVID-19. However, in cases of kidney failure, remdesivir administration should be considered only if the therapeutic benefits outweigh the risks because of concern about the accumulation of its solubilizing excipient sulfobutylether-beta-cyclodextrin and subsequent renal tubular injury or liver injury. Recently, reports from overseas indicating the safety of the use of remdesivir for COVID-19 patients on dialysis have been gathered.

**Case presentation:**

From June 2021, in our hospital, we started the administration of remdesivir to patients with moderate cases of COVID-19 undergoing hemodialysis, with careful consideration of the dosage and timing. Since then, six out of seven COVID-19 patients on hemodialysis who had received remdesivir have completely recovered. In a patient who died, the initial dose of remdesivir was administered after the case developed into severe COVID-19. All six patients who were able to start receiving remdesivir immediately at the stage of moderate COVID-19 recovered and were discharged without the need for mechanical ventilation. While, two out of four patients before May 2021 who had not been administered remdesivir at admission became severe, transferred to another tertiary hospital, and died. During and after remdesivir administration, no increase in serum transaminase to five times or more of the normal upper limit was observed in any of the cases. There were no other adverse drug reactions, such as infusion reaction, gastrointestinal symptoms, or anemia.

**Conclusions:**

We were able to administer remdesivir to six Japanese patients with moderate COVID-19 on hemodialysis safely. It is expected that the safe use of remdesivir will bring an increase in treatment options for moderate cases of COVID-19 in dialysis patients as well as subsequent improvement in treatment outcomes. However, to confirm the efficacy and safety of such use, further careful observation in more cases is required.

## Background

Since the end of 2019, novel coronavirus disease (COVID-19) has been a pandemic worldwide, and in Japan, over 1.3 million people have already been infected, and more than 15 thousand have died as of August 2021. Although the efficacy of vaccination is expected, it may need substantial time before the completion of herd immunity. Most chronic dialysis patients are elderly and have reduced immunity due to kidney failure; thus, they would be at very high risk of aggravation if they develop COVID-19 pneumonia. Therefore, in Japan, all COVID-19 patients on dialysis, regardless of severity, are indicated for hospitalization. At the end of August 2021, in Japan, according to a report by the COVID-19 Task Force Committee of the Japanese Association of Dialysis Physicians, the Japanese Society for Dialysis Therapy, and the Japanese Society of Nephrology, the mortality rate of COVID-19 patients on dialysis was as high as 16.6%, even considering that the mean age was high, compared to the rate of 1.2% of the entire population, which was reported by the Ministry of Health, Labor, and Welfare of the country.

In the treatment of COVID-19, only three drugs, remdesivir, dexamethasone, and baricitinib, had been approved by the Ministry of Health, Labor, and Welfare of Japan by the end of May 2021. Among these three drugs, remdesivir is the only antiviral drug, and the latter two are anti-inflammatory drugs. Since July 2021, the novel anti-severe acute respiratory syndrome coronavirus 2 (SARS-CoV-2) neutralizing antibody cocktail “casirivimab/imdevimab” has been available, but the antibody drug is indicated only for COVID-19 patients who are at high risk of aggravation and do not require oxygen administration.

In the general population, there are many reports on the effectiveness of remdesivir in moderate COVID-19 cases requiring oxygen administration. However, in cases with an estimated glomerular filtration rate (eGFR) below 30 mL/min/1.73 m^2^, remdesivir administration should be considered only if the therapeutic benefits outweigh the risks because of concern about the accumulation of its solubilizing excipient sulfobutylether-beta-cyclodextrin (SBECD) and subsequent renal tubular injury or liver injury. Thus, the treatment options for COVID-19 patients on dialysis who require oxygen administration are limited. In our hospital, until May 2021, we had refrained from administering remdesivir to COVID-19 patients on dialysis, and as a result, two of four of those cases had unfortunate outcomes. Consequently, effective and safe treatment strategies for such patients are urgently needed.

Recently, reports from overseas indicating the safety of the use of remdesivir for COVID-19 patients on dialysis have been gathered [1, 2]. Accordingly, we carefully started to administer remdesivir to patients with moderate cases of COVID-19 undergoing hemodialysis in our hospital in June 2021. Referring to the report by Aiswarya et al. [1], remdesivir 100 mg was intravenously infused (no initial loading) over a period of 2 h three times per week and five times in total so that the dosing would be completed 4 h before each hemodialysis session. We obtained written consent for the use of remdesivir with sufficient explanation. To avoid drug-induced liver injury, serum transaminase was measured before each dialysis session, and if the levels exceeded five times the upper limit of each normal range, it was planned to discontinue the administration of remdesivir immediately.

In our hospital, we used the classification of severity for COVID-19 presented by the Ministry of Health, Labor, and Welfare of Japan. In the classification, a mild case is defined as “percutaneous oxygen saturation (SpO_2_) of 96% or higher” and “no dyspnea or pneumonia on chest computed tomography (CT) scan is observed”, a moderate case I is defined as “SpO_2_ between 95 and 94%” or “dyspnea or pneumonia is observed, but the patient does not need oxygen administration”, a moderate case II is defined as “SpO_2_ of 93% or less” or “the patient needs oxygen administration”, and a severe case is defined as “the patient needs management in the intensive care unit or with mechanical ventilation”.

## Case presentation

We retrospectively observed all unvaccinated COVID-19 patients undergoing hemodialysis treated in our hospital between March 2021 and August 2021. The characteristics and clinical findings on admission and treatment outcomes in all 11 patients are described in Table 1. All three patients who died were over 70 years old, and the deceased patients tended to have high serum C-reactive protein (CRP) levels at admission.

**Table 1:** Characteristics and clinical findings on admission, and treatment outcomes

Case		Before May 2021				After June 2021						
		1	2	3	4	5	6	7	8	9	10	11
Gender	(M/F)	F	M	F	M	F	M	M	M	F	M	M
Age	(year)	70s	80s	70s	70s	70s	70s	40s	80s	50s	40s	60s
Dialysis vintage	(month)	336	63	5	7	21	252	0	307	26	24	27
Diabetes	(+) or (−)	(−)	(+)	(+)	(+)	(−)	(−)	(+)	(−)	(+)	(+)	(+)
Number of vaccinations	(time)	0	0	0	0	0	0	0	0	0	0	0
SpO2	(%)	82	95	95	95	89	92	95	84	95	88	94
Oxygen administration	(L/min)	0	0	0	0	0	0	0	0	0	0	0
Oxygen required to maintain SpO_2_ of 93% or higher (	L/min)	1	0	1	0	2	1	1	3	0	2	1
Severity of COVID-19		Moderate II	Moderate I	Moderate I	Moderate I	Moderate	Moderate II	Mild	Moderate II	Moderate I	Moderate II	Moderate I
Serum CRP	(mg/dL)	10.33	4.88	2.18	6.06	2.98	11.20	0.10	15.43	0.24	0.99	1.12
Serum LDH	(IU/L)	267	334	282	253	280	221	411	490	250	209	274
Serum ferritin	(ng/mL)	242	170	154	30	1445	73	110	251	270	139	65
Chest CT findings		Pneumonia	None	None	Pneumonia	Pneumonia	Pneumonia	Congestion	Pneumonia	None	Pneumonia	Pneumonia
When to start remdesivir administration		Not administered	Not administered	Not administered	After becoming severe and transferring to a tertiary hospital	At the diagnosis of moderate COVID-19	At the diagnosis of moderate COVID-19	At the diagnosis of moderate COVID-19	After becoming severe (O_2_ 5 L/min SpO_2_ 87%)	At the diagnosis of moderate COVID-19	At the diagnosis of moderate COVID-19	At the diagnosis of moderate COVID-19
Duration to discharge	(day)		25	10		13	18	15		11	12	21
Outcome		Transferred to a tertiary hospital and died	Discharged	Discharged	Transferred to a tertiary hospital and died	Discharged	Discharged	Discharged	Died	Discharged	Discharged	Discharged

*SpO*_*2*_ percutaneous oxygen saturation, *COVID-19* novel coronaviral disease, *CRP* C-reactive protein, *LDH* lactate dehydrogenase, *CT* computed tomography

In our hospital, remdesivir was generally not administered to COVID-19 patients undergoing hemodialysis until May 2021, and two of four patients with moderate COVID-19 rapidly developed into severe COVID-19, transferred to another tertiary hospital, and died (Table 1). One of the deceased patients received remdesivir and tocilizumab after transferring to the tertiary hospital, but he did not recover.

After June 2021, seven patients with moderate cases of COVID-19 have been administered remdesivir, and one of them has died (Table 1). In the patient who died, the first administration of remdesivir was 32 h after admission due to the timing of dialysis, and the respiratory condition rapidly became severe (SpO_2_ of 87% with 5 L/min of oxygen administration) 4 h before the initial administration of the drug. Of the six patients who were able to start receiving remdesivir immediately at the stage of moderate COVID-19, all completely recovered without the need for mechanical ventilation (Table 1), although three patients had worsening pneumonia on chest CT, and one of them received corticosteroid pulse therapy and tocilizumab.

However, due to the very small number of cases, a statistical analysis could not be conducted on the differences in clinical findings at admission and treatment outcomes including the number of days to discharge between the two groups.

No serious adverse drug reactions, such as infusion reaction, gastrointestinal symptoms, or anemia, were observed in any patients treated with remdesivir. Regarding liver injury, although a mild increase in serum transaminase was observed in one case, and the maximum values were less than five times the upper limit of each normal range (Table 2). Therefore, in the patient, remdesivir was administered in total of five times as scheduled, and after discharge, his serum transaminase normalized in the natural course. In other cases, no increase in serum transaminase above the normal upper limit was observed after drug administration (Table 2).

**Table 2:** Remdesivir administration and changes in serum transaminase levels

Case 5		▼			▼		▼		▼			▼	
Day	1	2	3	4	5	6	7	8	9	10	11	12	13
AST (U/L)	64	45		41	37		35					20	
ALT (U/L)	56	45		38	35		35					17	
Ag (pg/mL)	> 5000										70.65		

Case 6		▼		▼		▼			▼		▼						
Day	1	2	3	4	5	6	7	8	9	10	11	12	13	14	15	16	17
AST (U/L)	33	24		19		26			21		17	23			21		16
ALT (U/L)	42	32		24		30			26		19	18			10		9
Ag (pg/mL)	> 5000							> 5000			66.95						

Case 7				▼		▼			▼		▼		▼		
Day	1	2	3	4	5	6	7	8	9	10	11	12	13	14	15
AST (U/L)	18			18		21			21		20				11
ALT (U/L)	16			15		16			16		29				13
Ag (pg/mL)	> 5000														2.15

Case 9		▼			▼		▼		▼		
Day	1	2	3	4	5	6	7	8	9	10	11
AST (U/L)	19				8				12		
ALT (U/L)	18				12				16		
Ag (pg/mL)	> 5000										22.36

Case 10		▼			▼		▼		▼			▼
Day	1	2	3	4	5	6	7	8	9	10	11	12
AST (U/L)	21				18		24		33			61
ALT (U/L)	21				20		35		54			82
Ag (pg/mL)	> 5000											0.17

Case 11		▼		▼		▼			▼		▼										
Day	1	2	3	4	5	6	7	8	9	10	11	12	13	14	15	16	17	18	19	20	21
AST (U/L)	17	14		17		14			9		8		8		10			10			
ALT (U/L)	12	11		13		21			18		12		10		12			15			
Ag (pg/mL)	> 5000							270										0.66			

▼: remdesivir administration, AST: aspartate aminotransferase (reference value < 30 U/L), ALT: alanine aminotransferase (reference value < 35 U/L), Ag: severe acute respiratory syndrome coronavirus 2 antigen (reference value < 1.34 pg/mL)

We report in detail below the clinical course of six patients who were able to start receiving remdesivir immediately at the stage of moderate COVID-19.

### Case 5

A woman in her 70 s had a medical history of developing acute aortic dissection (Stanford A) 2 years prior, for which she underwent ascending aorta replacement and mitral valve replacement. As she also developed acute kidney injury at that time and her kidney function did not recover, she initiated maintenance hemodialysis. This time, she showed fever (37.7 °C) and decreased SpO_2_ (92%), and the polymerase chain reaction (PCR) test for SARS-CoV-2 was positive at a chronic care medical facility. With a diagnosis of COVID-19, she was transferred to our hospital.

On admission, since her SpO_2_ decreased to 89%, nasal oxygen support (2 L/min) was started. Then, the oxygen flow rate was adjusted to maintain an SpO_2_ of 93% or more. Chest CT scan showed ground-glass opacity in contact with the pleura on the upper lobe of the left lung. Blood tests showed slightly elevated serum hepatobiliary enzymes, such as aspartate aminotransferase (AST) 64 U/L, alanine aminotransferase (ALT) 56 U/L, and γ-glutamyl transpeptidase (γGTP) 54 U/L (Table 2). Serum bilirubin was within the normal range, and both hepatitis B surface (HBs) antigen and hepatitis C (HCV) antibody were negative. No morphological abnormalities in the liver and biliary tract were observed in abdominal CT scans. From the day of admission, similar to the treatment protocol for COVID-19 cases with preserved kidney function in our hospital, the anti-inflammatory drug dexamethasone 6.6 mg/day was intravenously infused, and heparin calcium 5000 units/day was subcutaneously injected to prevent thromboembolism. From the second hospital day, remdesivir 100 mg was administered by the method as we mentioned earlier. The fever disappeared the day after admission, and oxygen support was discontinued on the fifth hospital day. Dexamethasone was also discontinued on the seventh hospital day.

No increase in serum transaminase was observed after the start of remdesivir (Table 2). No other adverse reactions to remdesivir were observed. After meeting the discharge criteria issued by the Ministry of Health, Labor and Welfare of Japan, the patient returned to the previous medical facility on the 13th day.

### Case 6

A man in his 70 s had received hemodialysis for 21 years. The cause of kidney failure was neurogenic bladder due to spina bifida. This time, he showed fever, and the PCR test for SARS-CoV-2 was positive at a chronic care medical facility on the day before transfer to our hospital.

On admission to our hospital, the patient showed fever (39.0 °C) and decreased SpO_2_ (92%). Nasal oxygen support (1 L/min) was started immediately. On chest CT scan, ground-glass opacity was observed near the pleura on the dorsal side of the lower lobe of the right lung. From the day of admission, 6.6 mg/day dexamethasone was intravenously infused, and 10,000 units/day heparin calcium was subcutaneously injected. From the second hospital day, remdesivir infusion was started and administered a total of five times. On the second hospital day, the oxygen demand disappeared. On the fifth hospital day, the fever disappeared, and then intravenous dexamethasone was discontinued on the seventh day.

There was neither elevation of serum transaminase (Table 2) nor any other adverse drug reactions. On the 18th day, all these treatments were completed, and the next day, the patient returned to the previous medical facility.

### Case 7

A man in his 40 s who previously visited another hospital because of dyspnea urgently initiated maintenance hemodialysis due to end-stage kidney disease with type 2 diabetes 17 days before transfer to our hospital. In the previous hospital, a PCR test for SARS-CoV-2 was performed due to fever, and the result was positive. On the same day, a COVID-19 cluster occurred at that hospital, making it difficult to manage dialysis patients there, so the patient was transferred to our hospital.

When admitted to our hospital, the patient had no symptoms, his body temperature was 36.9 °C, and his SpO_2_ was maintained at 95% without oxygen support. Chest CT showed congestion in both hilar regions and a large amount of bilateral pleural effusion, but no apparent pneumonia was observed. The cardiothoracic ratio was 54.9% on chest X-ray, and marked pitting edema was found on both lower legs. His consciousness was clear, his blood pressure was 195/111 mmHg, his pulse rate was 93/min, his respiratory rate was 20/min, and his SARS-CoV-2 antigen level was > 5000 pg/mL. In the blood test, white blood cell (WBC) and CRP were within normal range at 7200/mm^3^ and 0.10 mg/dL, respectively. Plasma brain natriuretic peptide was as high as 2156.9 pg/mL.

Initially, we considered the patient to have a mild case of COVID-19 and did not administer remdesivir because there was no obvious finding suggestive of the presence of pneumonia. Although his body weight had been reduced from 94.0 to 79.2 kg in the previous hospital by a total of eight sessions of dialysis, we assessed that the slight decrease in SpO_2_ at transfer to our hospital had been due to residual body fluid excess and continued to remove the body fluid further by dialysis.

**Fig. 1:**
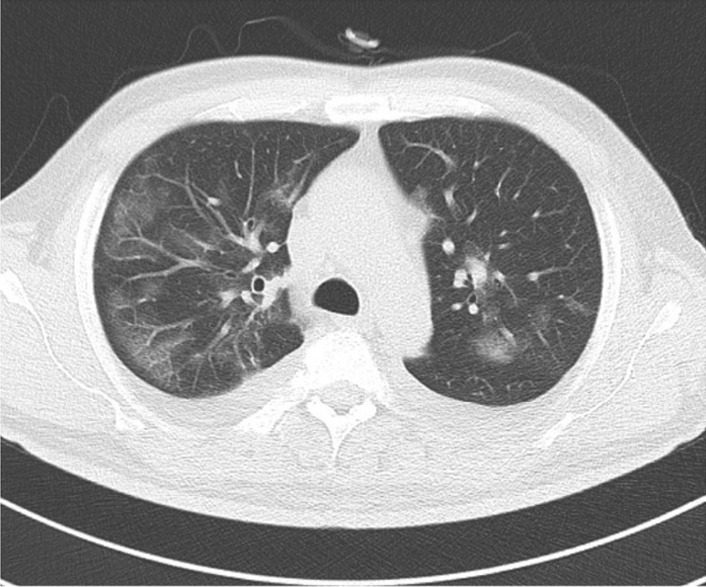
Chest CT image of pneumonia. (Case 7)

However, on the third hospital day, despite a further body fluid removal of 2.6 L after transfer, cough and dyspnea appeared. The patient’s body temperature increased rapidly to a maximum of 38.6 °C with chills, and the SpO_2_ dropped to less than 93%, requiring nasal oxygen support of 2 L/min Chest CT images showed that congestion and bilateral effusion were significantly reduced, but multiple new ground-glass opacities were observed mainly near the pleura in bilateral lung fields (Fig. 1). We diagnosed him with a moderate II case of COVID-19 that was at high risk for aggravation of pneumonia and thus started to administer intravenous remdesivir.

Anticoagulant therapy with heparin calcium was avoided because the patient’s left eye had been blind, his right eye had shown severe loss of visual acuity due to diabetic proliferative retinopathy, and the treatment had not been completed. Therefore, nafamostat mesylate was used as an anticoagulant during dialysis. For anti-inflammatory effects, dexamethasone 6 mg/day was orally administered.

During the clinical course, no adverse reactions to remdesivir were observed (Table 2). Fever was resolved on the seventh hospital day, dexamethasone was discontinued on the 10th day, and the oxygen demand disappeared on the 12th day. On the 13th day, remdesivir was discontinued with a total of five doses. In addition to COVID-19 treatment, the patient’s body fluid volume was also corrected by dialysis. On the 15th day, he returned to the previous hospital.

### Case 9

A woman in her 50 s with type 2 diabetes had been on dialysis for 2 years. She was admitted to our hospital because she had a cough for 2 days and a fever (37.3 °C) for 1 day and tested positive for SARS-CoV-2 PCR at a local clinic.

On admission, her body temperature was 37.8 °C, her SpO_2_ was 95% without oxygen administration, and her chest CT scan showed no apparent pneumonia. She began taking dexamethasone 6 mg/day and started an intravenous remdesivir infusion. During the clinical course, no adverse reactions to remdesivir were observed (Table 2). Heparin calcium was not administered because she was being treated for fundus hemorrhage due to diabetic retinopathy. Nafamostat mesylate was used as the anticoagulant during dialysis. On the sixth hospital day, a new, slight ground-glass opacity was observed on a chest CT scan, but no subjective symptoms or deterioration of SpO_2_ was observed, and the patient was discharged on the 11th day.

### Case 10

A man in his 40 s had been on dialysis for 2 years. He had type 2 diabetes, obesity (body mass index: 35.0 kg/m^2^), bronchial asthma, and sleep apnea syndrome requiring continuous positive airway pressure at night. He had developed fever (38.5 °C) and chills on the day before admission and had tested positive for SARS-CoV-2 PCR at a local clinic.

On admission, since the SpO_2_ was as low as 88%, oxygen administration at 2 L/min was started. Chest CT scan showed mild pneumonia. Based on those findings and comorbidities, the risk of aggravation was considered to be high. Subcutaneous heparin calcium 10,000 units/day and oral dexamethasone 6 mg/day were started on the first hospital day, and intravenous remdesivir infusion was started on the second hospital day. After that, although the serum AST and ALT had increased to 33 IU/L and 54 IU/L on the ninth hospital day, respectively, they did not exceed five times the upper limit of each normal range (Table 2), and remdesivir was administered five times as scheduled.

The patient’s pneumonia steadily resolved, and he was discharged from our hospital on the 12th day. At discharge, serum AST and ALT were as high as 61 IU/L and 82 IU/L, respectively (Table 2), but then spontaneously normalized to 18 IU/L and 16 IU/L, respectively, at his local dialysis clinic.

### Case 11

A man in his 60 s had been on dialysis for 2 years. He had type 2 diabetes, obesity (body mass index: 30.5 kg/m^2^), sleep apnea syndrome, secondary polycythemia, and angina with a history of percutaneous coronary intervention four times in total. He had developed fever (38.3 °C) 5 days before admission and had tested positive for SARS-CoV-2 PCR at his local dialysis clinic 3 days before admission. On the day of admission, while receiving hemodialysis at the clinic, not only fever, cough, and dysgeusia but also a drop to 94% in SpO_2_ were observed, and he was transferred to our hospital.

At transfer to our hospital, 1 L/min of oxygen administration was started. Chest CT scan showed mild pneumonia. Based on the patient’s comorbidities and past history, the risks of aggravation of pneumonia and development of thromboembolism were considered to be high. We immediately started to administer subcutaneous heparin calcium 10,000 units/day and oral dexamethasone 6 mg/day. Furthermore, in order to start remdesivir administration as soon as possible, we rescheduled hemodialysis 2 days earlier than the regular timing and started to infuse remdesivir on the second hospital day.

Despite those treatments, the SpO_2_ dropped further to 86% at 1 L/min of oxygen administration, and thus the oxygen flow rate was increased to 4 L/min on the seventh hospital day. Since chest CT scans showed a marked enlargement of ground-glass opacities on the eighth hospital day, corticosteroid pulse therapy (intravenous methyl-prednisolone 500 mg/day for 3 days) was performed between the ninth and 11th hospital days, and tocilizumab 800 mg (8.5 mg/kg) was infused on the 13th day.

Subsequently, the patient’s pneumonia rapidly improved, and oxygen administration was discontinued on the 18th day. Finally, he was discharged from our hospital on the 21st day without any adverse drug reactions including liver injury (Table 2).

## Discussion

Three therapeutic agents against COVID-19 were approved by the Ministry of Health, Labor, and Welfare of Japan by the end of May 2021: remdesivir, dexamethasone, and baricitinib. The latter two have anti-inflammatory effects and are expected to suppress cytokine storms associated with COVID-19, but among those approved drugs in Japan, only remdesivir is expected to have direct antiviral effects. Recently, the anti-SARS-CoV-2 neutralizing antibody casirivimab/imdevimab was added to the treatment options for COVID-19, especially in the early phase. However, the antibody drug is indicated only for patients who do not need oxygen administration. Other candidate agents include the antiviral drug favipiravir and the anti-inflammatory drug tocilizumab, but their effectiveness has not yet been proven.

Remdesivir is an adenosine nucleotide analog prodrug, and its metabolized active form competes with adenosine triphosphate (ATP) for incorporation into ribonucleic acid (RNA) and inhibits the action of viral RNA-dependent RNA polymerase. This results in the termination of RNA transcription and a decrease in viral RNA production [3]. The drug has been recognized as a promising antiviral agent against a wide array of RNA viruses, including SARS/middle east respiratory syndrome (MERS)-coronavirus, in cultured cells, mice, and nonhuman primate models [4].

Reports on the efficacy of remdesivir in human COVID-19 are also increasing. In a randomized, double-blind, placebo-controlled multicenter trial from China, although not statistically significant, patients receiving remdesivir (*n* = 158) had a numerically faster time to clinical improvement than those receiving placebo (*n* = 78) among patients with a symptom duration of 10 days or less [5]. In addition, another randomized controlled trial conducted in ten countries, including Europe, the United States, and Asia, showed that remdesivir was superior to placebo in shortening the time to recovery in adults who were hospitalized with COVID-19 and had evidence of lower respiratory tract infection (median recovery time 10 days vs. 15 days) [6]. However, the subgroup analysis did not show an effect in severe cases receiving high-flow oxygen therapy, mechanical ventilation, or extracorporeal membrane oxygenation (ECMO). In a meta-analysis of four randomized controlled trials including two previously mentioned trials (*n* = 7334), although patients given remdesivir were not associated with a significant reduction in the meantime to clinical improvement or mortality, they were more likely to demonstrate recovery and had higher rates of hospital discharge [7].

On the other hand, in mortality trials of four repurposed antiviral drugs, remdesivir, hydroxychloroquine, lopinavir, and interferon beta-1a (Solidarity Trial), recommended by World Health Organization expert groups, those four drugs had little or no effect on hospitalized patients with COVID-19, as indicated by overall mortality, initiation of ventilation, and duration of hospital stay [8].

It is possible that differences in the phase of remdesivir administration and disease severity between studies caused such discrepancies in outcome. In the clinical course of COVID-19, the main pathogenesis is considered to be virus proliferation several days after the onset and thereafter an excessive immune response [9]. Therefore, it is proposed to administer antiviral drugs mainly in moderate cases in the earlier phase and then anti-inflammatory drugs in severe or critical cases [10]. Accordingly, in Japan, the indication for the use of remdesivir has been expanded from “severe COVID-19 cases” to “patients with pneumonia due to SARS-CoV-2, including moderate cases”.

However, in cases with decreased kidney function, the excretion of SBECD, which solubilizes excipient remdesivir, would be delayed. Since there are concerns about subsequent liver injury or renal tubular injury due to its accumulation, it is stated that remdesivir administration in cases with eGFR below 30 mL/min/1.73 m^2^ should be considered only if the therapeutic benefits outweigh the risks.

SBECD is also included in intravenous polyconazole. It was reported that the half-life was 2.1 ± 0.4 h in normal subjects and 79.1 ± 24.5 h in dialysis patients on the interdialytic day and that 4-h hemodialysis shortened it to 5.0 ± 1.4 h [11].

According to the drug information on remdesivir published by the pharmaceutical company, 74% of it is excreted in the urine, the molecular weight is 602.58, and the protein binding rate is as high as 87.9, suggesting a low removal rate by dialysis. In adults with normal kidney function, the initial loading dose of remdesivir is 200 mg, followed by 100 mg from the second day. The median plasma half-life of the drug at 100 mg IV in healthy adult subjects is 0.96 h.

To remove SBECD by hemodialysis, Aiswarya D et al. administered remdesivir to hemodialysis patients 4 h before each dialysis session considering that 75–93% of remdesivir itself transfers to tissues in 4 h [1]. In their report, remdesivir 2.5 mg/kg (maximum: 100 mg) was administered up to six times for 21 moderate cases (43.8%) and 27 severe cases (56.2%), and there were no events of significant liver function test alterations [1]. Ultimately, one patient with a moderate case and nine patients with severe cases died. Remdesivir administration was started within 48 h of admission in 29 cases and after that in 19 cases; as a result, early administration of the drug shortened the duration of hospitalization by a mean of 5.5 days [1].

While, Thakare et al. reported that among 46 cases with kidney failure, including 16 end-stage kidney disease cases and 30 acute kidney injury cases, remdesivir administration at the same dose as for cases with normal kidney function, which was 200 mg initially and followed by 100 mg/day, induced a slight elevation in AST and/or ALT in three cases, but it did not exceed five times the upper limit of each normal range [2].

As mentioned above, since COVID-19 patients on dialysis have an extremely high risk of becoming severe, we started to administer the antiviral drug remdesivir for moderate cases of COVID-19 in June 2021. Since the report by Thakare et al. had included the cases with residual kidney function and the report by Aiswarya et al. had examined the dose setting of remdesivir for cases with kidney failure in more detail, we referred to the findings of Aiswarya et al. The dose of the drug was reduced, and it was carefully administered to avoid adverse drug reactions while considering the timing with dialysis. We also paid attention to physical findings, blood cell counts, and serum transaminase levels during and after administration of the drug. As a result, no significant adverse drug reactions occurred.

In conclusion, since June 2021, six out of seven COVID-19 patients on hemodialysis who received remdesivir have completely recovered. In particular, all six patients who started to receive remdesivir immediately at the stage of moderate COVID-19 recovered without serious adverse drug reactions. Considering that two out of four patients before May 2021, who had not been administered remdesivir at admission, became severe COVID-19 and died, it is expected that administration of the drug at the stage of moderate COVID-19 may be effective even in Japanese hemodialysis patients. Most recently, Kikuchi et al. reported in a nationwide cohort study of Japanese COVID-19 patients on dialysis that the mortality risk was significantly lower in patients who were treated with remdesivir [12]. Our findings are consistent with their results. While, in our observation, both of the two patients, who started receiving remdesivir after becoming severe COVID-19, got worse and died. We hope that future large-scale observational studies will also reveal the efficacy of remdesivir stratified by the phase of the drug administration and disease severity. Throughout further accumulation of knowledge on remdesivir administration to COVID-19 patients on dialysis, it is expected that the treatment options for those patients will increase and then the treatment outcomes will improve.

Although the present study is an observation of a very small number of cases and is thus insufficient for statistical analysis on the efficacy and safety of remdesivir in Japanese COVID-19 patients on dialysis, we consider the findings to be valuable information because it is the first report describing that no serious adverse drug reactions such as severe liver injury were found in a detailed observation of Japanese COVID-19 patients on dialysis receiving remdesivir. However, further large-scale observational studies are required to confirm the safety of remdesivir administration in those patients.

## Data Availability

All data supporting our findings are contained within this article.

## Abbreviations

COVID-19: Novel coronaviral disease
SARS-CoV-2: Severe acute respiratory syndrome coronavirus 2
eGFR: Estimated glomerular filtration rate
SBECD: Sulfobutylether-beta-cyclodextrin
SpO_2_: Percutaneous oxygen saturation
CT: Computed tomography
CRP: C-reactive protein
PCR: Polymerase chain reaction
AST: Aspartate aminotransferase
ALT: Alanine aminotransferase
γGTP: γ-Glutamyl transpeptidase
HBs: Hepatitis B surface
HCV: Hepatitis C virus
WBC: White blood cell
ATP: Adenosine triphosphate
RNA: Ribonucleic acid
MERS: Middle east respiratory syndrome
ECMO: Extracorporeal membrane oxygenation
